# Grafting Carbon Fibers with Graphene via a One-Pot Aryl Diazonium Reaction to Refine the Interface Performance of T1100-Grade CF/BMI Composites

**DOI:** 10.3390/ma17133288

**Published:** 2024-07-03

**Authors:** Weidong Li, Ziqi Duan, Mingchen Sun, Pengfei Shen, Huanzhi Yang, Xiangyu Zhong, Yang Zhang, Xiaolan Hu, Jianwen Bao

**Affiliations:** 1 National Key Laboratory of Advanced Composites, AVIC Composite Technology Center, AVIC Composite Corporation Ltd., Beijing 101300, China; liwdhappy@163.com (W.L.); dzq970920@163.com (Z.D.); 19821277882@163.com (P.S.); huanzhiyangavic@163.com (H.Y.); xyzhong2003@sohu.com (X.Z.); composite123@sina.com (Y.Z.); 2 School of Materials Science and Engineering, Beihang University, Beijing 100191, China; smc703@126.com; 3 College of Materials, Xiamen University, Xiamen 361005, China

**Keywords:** carbon fiber, graphene, surface modification, interfacial properties

## Abstract

In this study, a one-pot aryl diazonium reaction was used as a simple and mild method to graft graphene onto the smooth and inert surface of T1100-grade carbon fiber (CF) through covalent bonding without any damage on CF, to refine the interface performance of CF/bismaleimide (BMI) composites. XPS, SEM, AFM, and dynamic contact angle testing (DCAT) were used to characterize chemical activity, morphologies, and wettability on untreated and grafted CF surfaces. Meanwhile, the impact of the graft method on the tensile strength of CF was also examined using the monofilament tensile test. IFSS between CF grafted with graphene and BMI resin achieved 104.2 MPa after modification, increasing from 85.5 MPa by 21.8%, while the tensile strength did not decrease compared to the pristine CF. The mechanism of this interface enhancement might be better chemical bonding and mechanical interlock between CF grafted with graphene and BMI resin, which is generated from the high surface chemical activity and rough structure of graphene. This study may propose a simple and mild method to functionalize the CF surface and enhance the interface performance of composites without compromising the tensile properties of T1100-grade CF.

## 1. Introduction

In recent years, carbon fiber has been widely used in several fields, especially in aerospace, because of its outstanding properties such as excellent specific strength and modulus, good corrosion resistance, and temperature resistance. Composites with carbon fiber as reinforcement and polymer as matrix have become the preferred material in fields such as aerospace, shipbuilding, mechanical engineering, and the automotive industry [1,2,3]. In the aerospace industry, which involves the production of rockets, artificial satellites, and airplanes, carbon fiber-reinforced polymer composites (CFRPs) are widely applied in load-bearing structures, owing to their excellent specific strength and modulus. The application of CFRPs reduces weight and energy consumption, which can improve transportation capacity [4,5,6]. In order to satisfy the increasing performance requirements of advanced CFRPs, various high-performance CFs with higher tensile strength and modulus have been developed, such as T1000 and T1100 produced by Toray.

As a new generation of reinforcement, T1100 carbon fiber has extraordinary tensile strength and modulus. However, due to the severe chemical composition and smooth surface of T1100, the wettability and adhesion between CF and resin are limited, causing a weak interfacial bond, which negatively affects the performance of CFRPs [7,8,9]. Therefore, researchers have used different approaches such as chemical grafting [10,11], chemical oxidation [12], high-energy irradiation [13], plasma treatment [14,15], and nanoparticles [16,17,18,19,20,21,22,23,24,25,26] to achieve the goal of enhancing the interface performance of CFRPs. In recent years, with the development and application of nanomaterials, modifying the surface of CFs with nanoparticles such as graphene, nanometer SiO_2_, and carbon nanotubes (CNTs) to improve the interface performance of CFRPs has become a hot topic [17,18,19]. 

As is well known, graphene has outstanding mechanical and electrical properties, as well as ultra-low thickness, providing a large specific surface area. In recent research, it has been shown that graphene can be applied to enhance the interface performance of CFRP. Dong [20] used nanocoating technology to achieve the doping of graphene nanosheets onto the interface area of CFRPs, resulting in a 44.4% increase in the interfacial shear strength (IFSS) of modified CF/epoxy. Deng et al. [21] revealed a remarkable increase of 55.6% in the IFSS of CFRPs by uniformly grafting a thick layer of graphene onto the CF surface through electrophoretic deposition methods. Zhang [22] grafted amino-functionalized graphene on the flat surface of CF through direct chemical bonding, which exhibited a great improvement in the IFSS of CFRPs. The DCAT and XPS results showed that grafting amino-functionalized graphene significantly improved the surface energy and active functional groups of the CF surface. He et al. [23] introduced graphene onto the CF surface by chemical vapor deposition, which improved the wettability and roughness of the CF surface, resulting in a significant increase in the ILSS of CF/PTFE composites from 49.45 MPa to 71.91 MPa. Jiang [24] utilized graphene to significantly improve the interface performance of CF/epoxy composites, which ultimately led to a significant improvement in the surface energy of CF and a 55.6% increase in the IFSS of the composites. However, the modification methods used in the above studies are relatively complex, with poor controllability, and may cause damage to CF.

An aryl diazonium reaction has the advantages of simple operation, good controllability, and mild conditions, which can modify the CF surface without damaging the properties of CFs. Therefore, it has become a popular method for improving the interface of CFRPs. Many researchers used different nitrous acid compounds, such as t-Bu-ONO, NaNO_2_, and NBu_4_BF_4_ [25,26,27], for aryl diazonium reactions to directly graft aniline polymers onto the CF surface, improving the interfacial bonding of CFRPs. Although the aryl diazonium reaction can facilitate the formation of polymers on the CF surface, its molecular chains are small with a relatively slight improvement effect on the CF surface. Thus, using an aryl diazonium reaction as a bridge to introduce nanoparticles onto the CF surface becomes a feasible method to enhance the interface performance of CFRPs. Wang [28] used a two-step aryl diazonium salt reaction, which generated aniline groups on the CF surface, and introduced amino-functionalized CNTs on the CF surface to achieve a significant improvement in the interface performance of the composite. Liu [29] carried out the grafting of CNTs using a step-wise reduction of the diazonium salt generated in situ on the CF surface, significantly improving the interphase of CF/epoxy. Sun [30] and Chen [31] carried out aryl diazonium salt reactions through electrochemical methods, introducing CNTs and MXene onto CF surfaces, which were beneficial for the interface performance of CF/PEEK and CF/epoxy, respectively. Wu [32] used isopentyl nitrite instead of sodium nitrite for the one-pot aryl diazonium reaction under heating and stirring, successfully achieving the grafting of CNTs, which improved the surface roughness and chemical activity of CF. All the studies above show the simplicity, controllability, and efficiency of this grafting method.

This article used a simple and effective method to graft graphene onto the CF surface using a one-pot aryl diazonium reaction under a temperate process environment in the form of covalent bonding, achieving the goal of improving interfacial properties. In this study, the chemical and physical properties of the CF surface modified with different concentrations of graphene were systematically investigated. Meanwhile, the influence of graphene modification on the interfacial performance of CF/BMI was investigated. Based on the research results, a detailed analysis and discussion were conducted on the interface-strengthening mechanism of graphene-modified CFs.

## 2. Materials and Methods

### 2.1. Materials

The CF samples (T1100 grade) were purchased from Weihai Tuozhan Industries Inc., Weihai, China. The mechanical properties of T1100 CF used in this article were tested according to ASTM D 4018 [33], and the results are shown in Table 1. Before modification, CF was de-sized using the Soxhlet method, and the obtained samples labeled as CF were dried at 100℃ for 3 h. The resin used in this article was high-toughness bismaleimide resin marked as BMI provided by AVIC Composite Corporation Ltd., Beijing, China. Graphene was purchased from Chengdu Organic Chemistry Corporation Ltd., Chengdu, China. Acetone, isopentyl nitrite (90%, AR), P-phenylenediamine (97%, AR), and N, N-dimethylformamide (99.5%, AR) were purchased from Shanghai Aladdin Biochemical Technology Corporation Ltd., Shanghai, China. 

### 2.2. Preparation of CF-G

**Figure 1:**
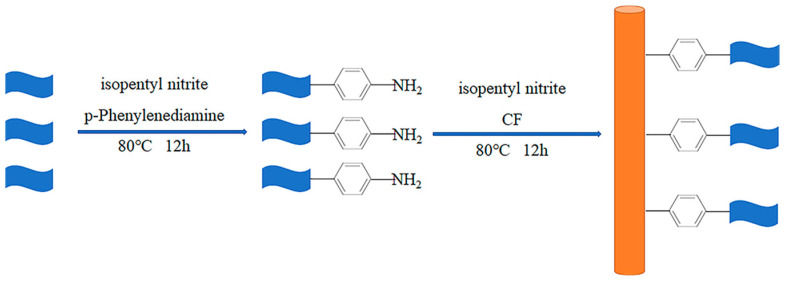
Schematic sketch of preparation for CF-G.

p-Phenylenediamine (2.0 g) and isoamyl nitrite (0.8 mL) were added to deionized water (100 mL) and mixed well; then, the mixed solution was poured into a three-necked flask. Graphene (6.0 g) was added to the flask, and the mixture was stirred for 12 h at 80 °C to obtain graphene grafted with amino groups (graphene–NH_2_). In order to introduce graphene onto the CF surface, a different amount of graphene–NH_2_ was dispersed in deionized water first via a 60 min ultrasonic treatment to obtain graphene–NH_2_ solutions with different concentrations (0.5 wt%, 1.0 wt%, and 1.5 wt%). Then, a bundle of CF was wrapped around a frame and added to a three-necked flask together with a graphene–NH_2_ solution (100 mL) and isoamyl nitrite (1.0 mL). The frame wrapped with CF was placed flat in the three-necked flask, and then the magnetic stirring rod was put under the frame. Afterward, the three-necked flask was stirred vigorously at 80 °C for 12 h. Lastly, DMF and deionized water were used to wash the fiber grafted with graphene (denoted as CF-G) multiple times until the color in the waste liquid faded; then, the fibers were dried. CF-G prepared using the graphene–NH_2_ solution with various concentrations are labeled as CF-G-0.5, CF-G-1.0, and CF-G-1.5. Figure 1 shows the schematic sketch of the preparation of CF-G.

### 2.3. Characterization

SEM (JEOL JSM-7500, Tokyo, Japan) was used to examine surface morphology to determine the distribution of graphene on the CF surface with different concentrations of graphene.

Surface morphology and roughness were tested using AFM (Bruker Dimension icon, Billerica, MA, USA). The scanning range was set to 3 × 3 μm with a scan frequency of 1 Hz. NanoScope Analysis 1.7 software was used for roughness calculation. Three sets of data were calculated for each type of CF.

The X-ray photoelectron spectrometer analyzer model EscaLab 220i-XL (Thermo Fisher Company, Waltham, MA, USA) was used to test the chemical elements and functional groups on the CF surface. The radiation source was Al K (1456.6 eV). Then, 1–2 cm of carbon fiber tow was taken and pasted on the special sample stage of XPS for testing.

A dynamic contact angle meter (Dataphysics DCAT25, Filderstadt, Germany) was used to characterize the wettability of the CF surface. Each type of CF sample was tested 3 times, and a single experiment used 5 carbon fiber monofilaments.

A universal mechanical testing machine (Instron 5967, Boston, MA, USA) was used to measure the single filament tensile strength of CF, with testing standard referring to GB/T 31290 [34] (National Standard of China) Speed glue was applied to stick CF monofilaments onto both ends of the paper strip, with a gauge distance of 25 mm. The back paper strip was cut off at the beginning of the test, with the loading speed set as 2 mm/min. For every CF obtained in this work, at least 30 sets of samples had to be tested, and the monofilament tensile strength was calculated through a bivariate Weibull distribution.

A self-made microsphere debonding device was used to examine the *IFSS* between different CF and BMI. Double-sided tape was utilized to stick a single filament onto a C-shaped metal frame, and a small amount of resin was dipped into the fixed single filament with a needle. The monofilament with resin microspheres was placed in the oven and was heated according to the curing process of BMI used in this study (180 °C for 2 h and 210 °C for 6 h). After the samples were completed, the resin microspheres were clamped between two blades, and a force was applied to the CF to pull the fibers at 0.05 mm/min. *IFSS* is determined using the following equation [35]:

(1) $$IFSS=\frac{F_{max}}{\pi dl}$$

where *F_max_* denotes the maximum shear force during testing, *d* denotes the CF diameter, and *l* denotes the length covered by the microsphere. At least 10 sets of data were obtained for each type of CF.

## 3. Results and Discussion

### 3.1. Characterization of Graphene-NH_2_

**Figure 2:**
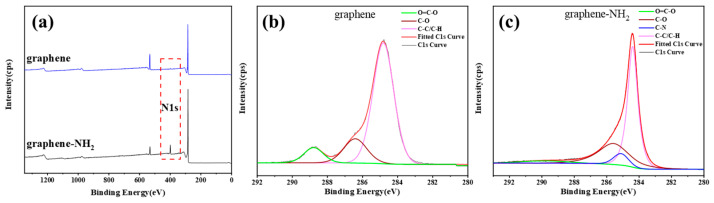
(**a**) The wide-scan XPS spectra: C1s of (**b**) pristine graphene and (**c**) graphene–NH_2_.

XPS was adopted to investigate the chemical composition of graphene–NH_2_ and the spectra of wide-scan XPS and high-resolution C1s are compiled in Figure 2. Compared with untreated graphene (Figure 2a,b), a significant N1s peak at 399.28 eV (Figure 2a) and C-N bond peak at 285.4 eV (Figure 2c) can be clearly observed, indicating that the amino groups were introduced onto graphene successfully.

### 3.2. Surface Morphologies and Properties 

**Figure 3:**
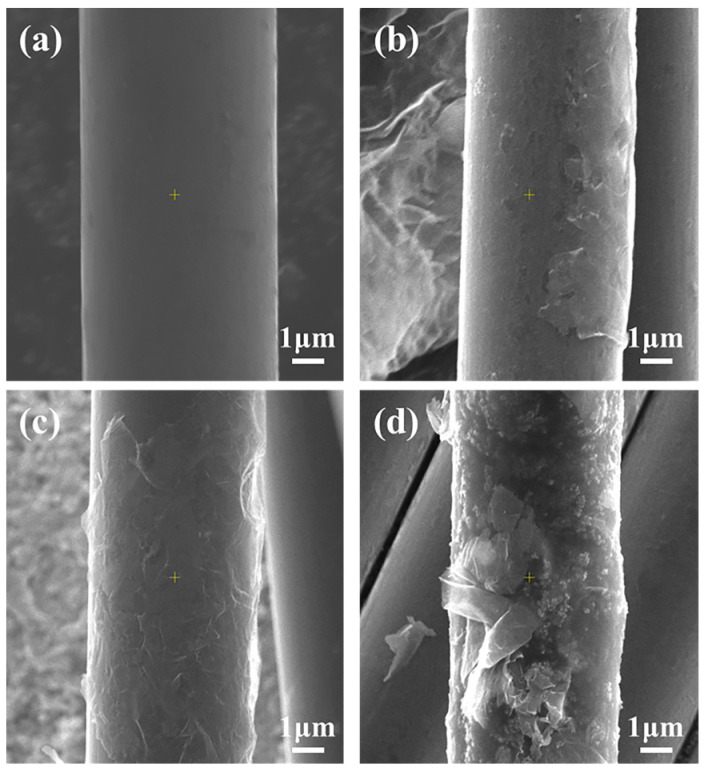
SEM images of CF surface before and after grafting: (**a**) CF; (**b**) CF-G-0.5; (**c**) CF-G-1.0; (**d**) CF-G-1.5.

The surface morphologies of pristine and graphene-modified CF are shown in Figure 3. As shown in Figure 3a, the CF surface without graphene is smooth, with fewer defects such as grooves and wrinkles, and therefore the physical friction between the CF and BMI resin is weak, which is averse to the interfacial integration between BMI and CF, thereby adversely affecting performance and toughness of the CFRP. After being combined with different grafting densities of graphene (Figure 3b–d), the smooth and neat surface of CF is covered to varying degrees with graphene nanoparticles.

The morphology of graphene on the CF surface changes with an increase in the graphene concentration. When the concentration of graphene is 0.5 wt% (Figure 3b), there is less graphene grafted on the CF surface, and nanosheets cannot cover the entire surface of CF. With the concentration of graphene increasing to 1.0 wt% (Figure 3c), the grafted graphene uniformly covers the surface of CF. Considering CF-G-1.5 (Figure 3d), excessive graphene is dispersed on the CF surface, with noticeable agglomeration occurring.

**Figure 4:**
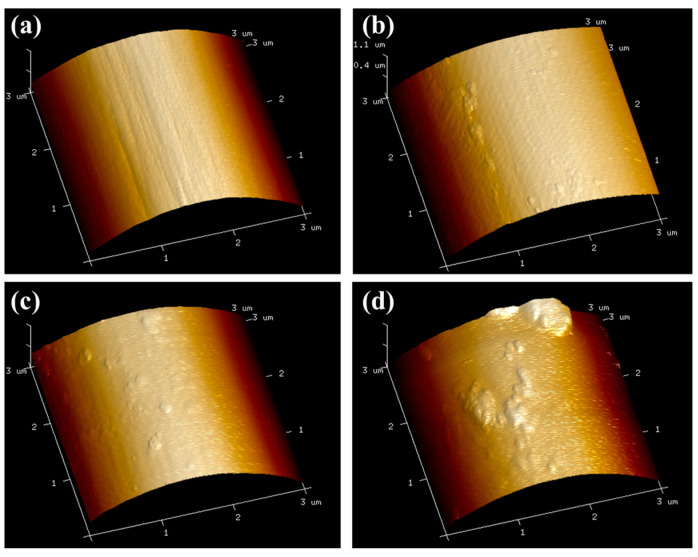
AFM images of CF surface before and after grafting: (**a**) CF; (**b**) CF-G-0.5; (**c**) CF-G-1.0; (**d**) CF-G-1.5.

In this study, AFM was also utilized to characterize different CF surface morphologies, as shown in Figure 4. AFM and SEM complement each other, thus depicting the morphologies of CF surface more accurately. The results of different levels of CF surface roughness are shown in Table 2. The pristine CF surface is mostly smoother, presenting a roughness of 5.7 nm. Compared with the pristine CF, dispersed graphene is observed after the introduction of graphene, as shown in Figure 4b–d. The results indicate that more graphene is present on the surface of CF, leading to a sharp increase in the surface roughness. As shown in Figure 4b, a slight amount of graphene is distributed unevenly on the CF surface. With the increase in graphene from 0.5 wt% to 1 wt%, a homogeneous distribution of graphene can be observed, resulting in an increase in roughness to 16.7 nm, as seen in Figure 4c. However, for CF-G-1.5, some graphene agglomerates together, leading to the formation of oversized bumps, as shown in Figure 4d, with the roughness further improved to 19.7 nm.

With the introduction of graphene as a connection between resin and CF into the interphase, surface roughness increases, which can enhance the physical meshing between resin and CF. During the process of fiber extraction from the matrix, greater friction is generated, which is beneficial for improving the reinforcement effect of CF on the composite [36].

### 3.3. Surface Chemical Characteristics of CF

**Figure 5:**
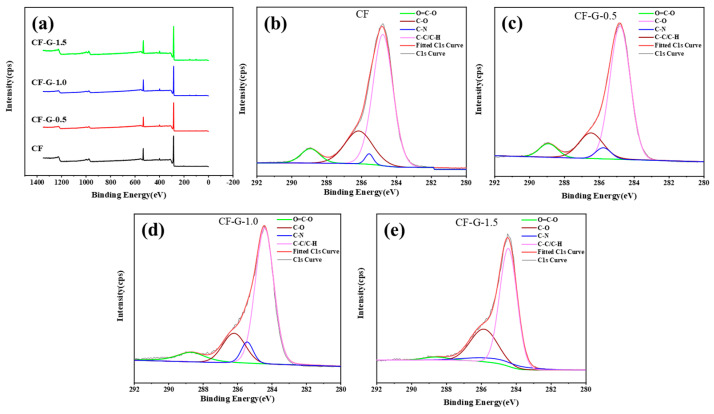
(**a**) Wide-scan spectra of CF and CF-G. High-resolution C1s spectra of (**b**) CF, (**c**) CF-G-0.5, (**d**) CF-G-1.0, and (**e**) CF-G-1.5.

The element composition of CF and CF-G was studied by XPS. The relative atomic content of different CF surfaces is shown in Table 3, and the wide-scan XPS spectrum is shown in Figure 5a. In order to accurately investigate the ratio of different functional groups of different CF surfaces, the C1s peak of XPS was fitted and analyzed, as shown in Figure 5b–d. Relative concentrations of functional groups are listed in Table 4. From the results, it can be observed that the CF surface before and after introduction is mainly composed of C, O, and N. With the introduction of graphene, the ratio of O atoms to C atoms on the CF surface increased from 0.137 to 0.163. At the same time, the introduction of amino groups led to an increase in the content of N atoms and the proportion of -C-N-. Compared to untreated CF, the proportion of active functional groups, for instance, -N-C- -O-C- and O=C-O-, increased from 27.66% to 32.94% after introducing graphene. The grafting of graphene helped to transform the inert surface of CF into a polar and active surface, thereby improving CF surface energy and the wettability of resin to CF.

### 3.4. Wettability of CF

**Figure 6:**
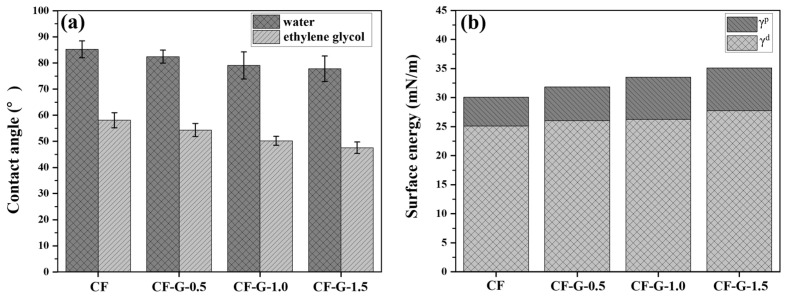
(**a**) Contact angle and (**b**) surface energy of CF surfaces.

The wettability of resin on the CF surface is crucial for the interface performance of CFRPs. Typically, excellent wettability enhances the integration between resin and CF surface, thereby improving the interfacial performance. The contact angles between the test liquid (deionized water and ethylene glycol) and CF were utilized to assess the surface energy of CF, as depicted in Figure 6a. Surface energy, including polar and dispersive components (γ^p^ and γ^d^), was calculated, and the results are illustrated in Figure 6b. The CF grafted with graphene exhibited reduced contact angles with water and ethylene glycol compared to untreated CF. Notably, CF-G-1.5 had the smallest contact angle with the two liquids, decreasing from 85.3° and 58.1° to 77.7° and 47.5°, respectively. Additionally, the modification of the CF surface improved the surface energy to varying degrees. Significantly, CF-G-1.5 had the highest surface energy, increasing from 30.0 to 35.1 mN/m while simultaneously increasing γ^p^ from 4.9 to 7.4 mN/m. This is because grafting graphene, which possesses good surface polarity and activity, onto CF enhances the wettability of the polar resin matrix on the CF surface. This improvement is owing to the abundance of active functional groups of graphene, leading to an increase in its polar components. At a low concentration of 0.5 wt%, graphene could not fully coat the entire CF surface, resulting in a limited increase in its polar components. When the graphene concentration reached 1 wt%, the graphene nanosheets uniformly coated the entire CF surface, with a further increase in polar components. As the graphene concentration continued to rise to 1.5 wt%, excessive concentration led to its aggregation on the CF surface (Figure 3d), without significantly increasing its surface energy [37]. In summary, the grafted CF exhibited higher surface energy and effectively enhanced the wettability of resin on CF.

### 3.5. Tensile Properties of Monofilament

**Figure 7:**
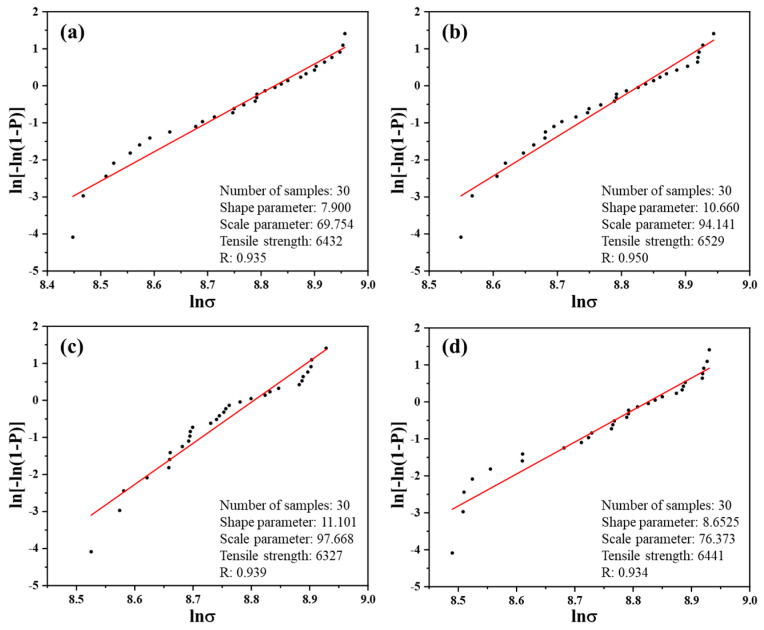
Weibull distribution fitting curves of monofilament strength: (**a**) CF; (**b**) CF-G-0.5; (**c**) CF-G-1.0; (**d**) CF-G-1.5.

As a key role in CFRPs, the strength of CF significantly impacts the performance of composites. To explore the influence of the surface modification method used in this study on CF mechanical performance, the monofilament tensile strength before and after modification was characterized, with the results and Weibull distribution depicted in Table 5 and Figure 7. The figures indicate that the monofilament tensile strength of pristine CF was 6432 MPa, while those of modified CFs were 6529 MPa, 6327 MPa, and 6441 MPa, respectively. In comparison with the strength of untreated monofilament, the variation in tensile strength after modification is within 3.2%, which can be considered the same. In contrast with the traditional strong acid oxidation process, the reaction conditions of surface treatment used in this research are relatively mild. While enhancing the surface characteristics of CF, the damage to its surface structure can be completely ignored.

### 3.6. IFSS of CF/BMI Composites

**Figure 8:**
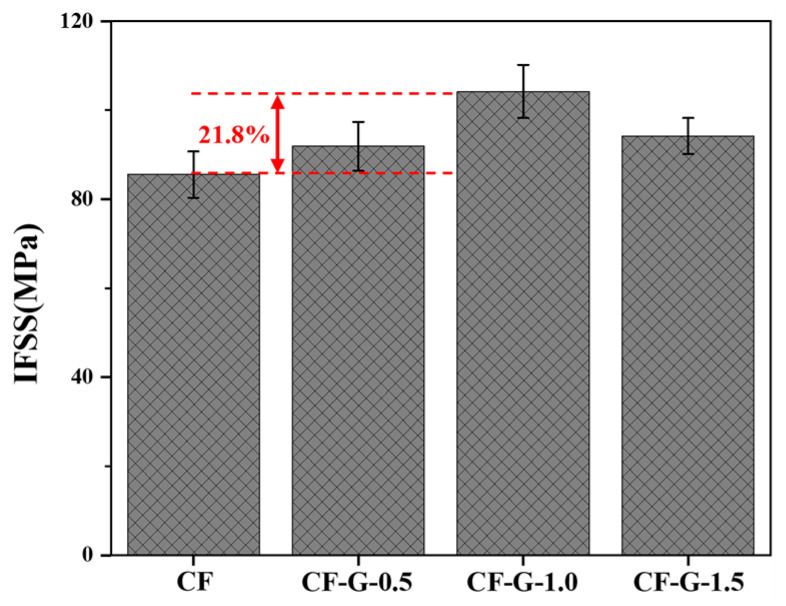
IFSS of different CF/BMI composites.

The microsphere debonding test is generally used to characterize the IFSS between resin and CF, reflecting the interface performance of CFRPs at the microscale. In order to evaluate the improvement in graphene modification on interface performance, the IFSS between CFs and BMI resin was tested, as shown in Figure 8. It can be observed that the IFSS value between untreated CF and BMI was 85.5 MPa, which was the lowest among all CFs, due to the weak interface bonding between CF and BMI caused by its smooth and inert surface. With graphene grafted on the CF surface, the IFSS improved to varying degrees. After grafting, the highest IFSS reached 104.2 MPa, which increased by 21.8% compared to pristine CF. The analysis of the test results revealed the following two points: Firstly, graphene coating on the smooth CF surface (Figure 2c) promotes surface roughness and improves the physical interlock between BMI and CF. Secondly, the abundant polar functional groups like amino and oxygen-containing groups on the graphene surface increase the surface energy, which is beneficial for the wettability of BMI resin to CF. Meanwhile, unreacted amino groups on the graphene surface participate in resin-curing reactions, forming a strong chemical combination between CF and BMI resin. The above factors are involved in the enhancement of the IFSS between BMI and CF. As the graphene content increased from 0.5 wt% to 1.5 wt%, the IFSS between CF and BMI first increased and then decreased at the content of 1.5 wt%. With the content increasing, the state of graphene on the CF surface gradually transitioned from partial adhesion to complete coating. Continuing to increase the content led to the excessive aggregation of graphene on the CF surface, which resulted in graphene nanosheets folding and stacking and thus a decrease in interfacial properties.

**Figure 9:**
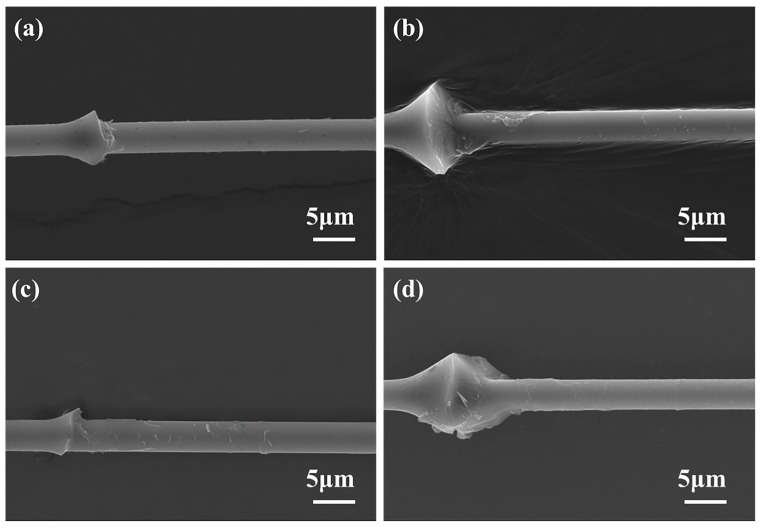
SEM images of fracture morphology after debonding: (**a**) CF; (**b**) CF-G-0.5; (**c**) CF-G-1.0; (**d**) CF-G-1.5.

**Figure 10:**
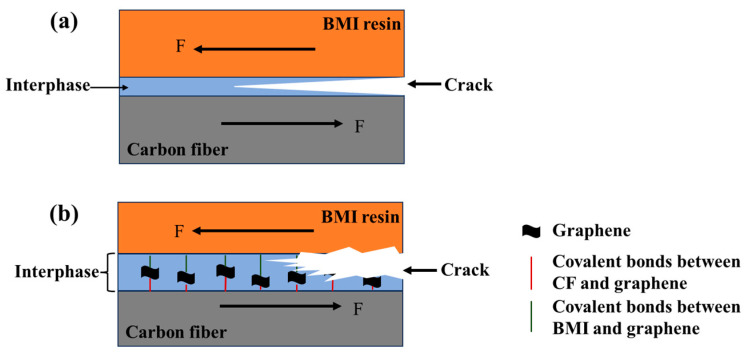
Schematic diagram of crack propagation: (**a**) CF/BMI; (**b**) CF-G-1.0/BMI.

To explain the additional reason why grafting graphene on the CF surface improves the CF/BMI interface properties, SEM was used to observe the surface condition of CF after resin microsphere peeling, the results of which are shown in Figure 9. From the figure, it can be inferred that after microsphere debonds from the untreated CF, the surface is smooth without residual BMI resin. This is because the pristine CF surface is flat and non-polar, with frail Van der Waals force, and therefore the interphase is prone to delaminate. Figure 9b–d show the surface morphologies of CF after introducing graphene. Considering CF-G-1.0, there is massive BMI resin residue on the CF surface (Figure 9c), indicating that BMI resin adheres to graphene nanosheets on the CF surface, which generates strong interfacial adhesion between BMI and CF. As the graphene content increased, there was an increase in residual resin on the CF surface, indicating an improvement in the bonding force between CF and resin. The surface morphology for when the concentration increased to 1.5 wt% is shown in Figure 9d, from which it can be observed that some remaining graphene is present on the CF surface with less residual resin. Excessive graphene led to a tendency to aggregate on the CF surface, causing thick graphene layers with a weak bonding force between each other. When microsphere debonding occurred, resin and topside graphene were peeled off together, resulting in a smooth CF surface without the remaining BMI fragments. In summary, the mechanism of interface strengthening between graphene-grafted CF and BMI mainly includes the following aspects (Figure 10) [31]: Firstly, the surface energy of CF grafted with graphene is effectively improved because of rich active functional groups on the graphene surface, which is beneficial for the wettability of resin on the CF surface, thereby generating good interface integration. Secondly, the graphene layer on the CF surface can promote surface roughness, which can promote the physical interlock with BMI and suppress the propagation of microcracks, as seen in Figure 10. Thirdly, unreacted amino and oxygen-containing functional groups can react with BMI monomer during curing, forming chemical bonds between CF and BMI resin (Figure 10b). In summary, interface integration is greatly enhanced after graphene grafting, resulting in a high IFSS.

## 4. Conclusions

In summary, graphene was successfully introduced to the CF surface using a one-pot aryl diazonium reaction, which improved the interface performance between CF and BMI. The results of SEM, AFM, and XPS suggest that the grafting of graphene promotes the chemical activity and surface roughness of CF, resulting in the enhancement in the surface energy of CF after grafting, which is conducive to the sufficient wetting of BMI resin on the CF surface. As a result, the IFSS of graphene-modified CF improved from 85.5 MPa to 104.2 MPa, indicating an increase of 21.8%, which means a significant improvement in the CF/BMI interface. At the same time, this modification method did not have a negative impact on the mechanical performance of CF. Considering the mechanism of interface enhancement, it was found that the introduction of graphene improved the surface activity and roughness of CF, resulting in better chemical integration and mechanical interlocking between BMI and CF, which enhanced interfacial bonding. Meanwhile, the presence of graphene nanosheets suppressed microcrack propagation in the interface region, significantly improving the interface performance of CF/BMI composites. In this work, the one-pot aryl diazonium reaction proved to be a simple, mild, and efficient approach to grafting graphene onto the CF surface, providing a novel attempt to modify CF in the future.

## Figures and Tables

**Table 1:** Mechanical properties of T1100 CF used in this study.

Properties	Tensile Strength (MPa)	Elastic Modulus (GPa)	Elongation (%)
Value	6663	342	1.93

**Table 2:** The results of roughness of CF surface before and after grafting.

Samples	CF	CF-G-0.5	CF-G-1.0	CF-G-1.5
R_q_/nm	5.7 ± 1.4	10.2 ± 1.4	16.7 ± 2.3	19.7 ± 1.5
R_a_/nm	4.4 ± 1.0	7.28 ± 0.8	13.7 ± 1.8	15.7 ± 2.5

**Table 3:** Relative atom content of different carbon fiber surfaces.

Sample	Relative Content of Atom (%)			O/C	N/C
	C1s	O1s	N1s		
CF	85.82	11.73	2.45	0.137	0.028
CF-G-0.5	84.39	12.82	2.79	0.152	0.033
CF-G-1.0	83.36	13.59	3.05	0.163	0.037
CF-G-1.5	83.46	12.87	3.67	0.154	0.044

**Table 4:** The results of high-resolution XPS spectra of C1s.

Samples	-C-C-/-C-H		-C-O-		-O-C=O		-C-N-		Active Functional Groups Ratio (%)
	B.E. (eV)	P.C. (%)	B.E. (eV)	P.C. (%)	B.E. (eV)	P.C. (%)	B.E. (eV)	P.C. (%)	
CF	284.8	72.34	286.4	18.55	288.7	6.96	285.7	2.15	27.66
CF-G-0.5	284.8	69.93	286.5	17.31	288.7	7.88	285.7	4.88	30.07
CF-G-1.0	284.8	67.91	286.4	17.53	288.7	8.33	285.7	6.23	32.09
CF-G-1.5	284.6	67.06	286.3	20.77	288.7	4.41	285.6	7.76	32.94

**Table 5:** The results of monofilament tensile test.

Carbon Fiber	Number of Samples	Shape Parameter	Scale Parameter	Tensile Strength/MPa	R
CF	30	7.900	69.754	6432	0.935
CF-G-0.5	30	10.660	94.141	6529	0.950
CF-G-1.0	30	11.101	97.668	6327	0.939
CF-G-1.5	30	8.653	76.373	6441	0.934

## Data Availability

Data are contained within the article.
